# Trend Factor Smoothing and Tasmanian Devil Optimization based Siamese Neural Network for anomaly detection in predictive maintenance

**DOI:** 10.1038/s41598-025-25312-w

**Published:** 2025-11-24

**Authors:** Ida Hector, Rukmani Panjanathan

**Affiliations:** School of Computer Science and Engineering, Vellore Institute of Technology, Chennai, Tamil Nadu 600127 India

**Keywords:** Anomaly detection, Cyber physical systems, Predictive maintenance, Deep learning, Optimization, Engineering, Mathematics and computing

## Abstract

In today’s business world, predictive maintenance is essential since it helps organizations prevent equipment breakdowns and minimize downtime. A novel technique that employs machine learning to anticipate equipment failures is anomaly detection-based predictive maintenance. This method helps maintenance teams to foresee and prevent problems by looking for patterns and anomalies in historical data. This lowers the possibility of unplanned downtime and boosts overall productivity. Using optimized deep learning, this study is intended to create an advanced deep learning model for anomaly detection in the predictive maintenance of cyber-physical systems, incorporating trend factor smoothing with Tasmanian devil optimization (TFsTDO) and siamese neural networks (SNN) to enhance detection precision and operational efficacy. The proposed TFsTDO-SNN system encompasses preprocessing through Box–Cox transformation, feature selection utilizing an innovative TFsTDO algorithm, anomaly injection via oversampling, and anomaly detection employing an SNN trained by TFsTDO. The model was assessed utilizing the CNC Mill Tool Wear dataset, with performance criteria comprising precision, recall, and F1-score. Experimental findings indicate that TFsTDO-SNN attains a precision of 96.5%, a recall of 97.2%, and an F1-score of 96.9%, surpassing traditional approaches including GA, AdaBoost, LSTM-autoencoder, and CNN-LSTM. The TFsTDO-SNN model provides a strong solution for anomaly identification in predictive maintenance, with prospects for future improvements using explainable AI and sophisticated optimization methods.

## Introduction

Many sectors are confronting both new opportunities and challenges in preserving their competency and meeting market demands as a result of speedy growth of communication and information technologies and incorporation of advanced analytics into products, services and manufacturing^1^. This kind of integration, known as CPSs, dramatically alters the manufacturing process, maintenance methods and management by fusing strengths of internet technologies with optimal industrial manufacturing^2^. The ability of CPSs to connect physical devices and infrastructure, like intelligent power grids and self-driving cars, is an essential feature. These devices can be used for a variety of intelligent applications, including Industry 4.0 technologies, intelligent manufacturing facilities, the energy sector, and advanced transportation systems. CPSs are the next generation of intelligent systems that use modern computer, communication, and control technologies to improve real-world operational systems’ efficacy, reliability, security, and other performance metrics. Presently, a variety of scholars, governmental policymakers, technical personnel, and industry professionals are actively engaged in the advancement of CPSs in response to the proliferation of multiple applications conforming to global standards and crucial infrastructures for the evolution of smart urban areas. Moreover, defense authorities in different nations heavily rely on the standardized progression of CPSs, given that defense mechanisms such as naval fleets, groupings of unmanned vehicles, and unmanned aerial vehicles are indispensable components in the realm of CPSs^3^.

The increasing complexity of CPSs causes systems to become increasingly varied and complex, making technical upkeep a challenging issue. In the context of Industry 4.0, where an IoT platform acts as a monitoring and decision-making system, intelligent PHM solutions, or predictive maintenance, are demonstrating potential application capabilities and gaining acceptance swiftly to address the maintenance challenge of CPSs^2^. For industries, machine breakdowns can be highly costly since they cause unscheduled downtime and lost productivity. Regular, regular maintenance is how most maintenance programs minimize machine downtime^4^. It includes the technological steps necessary to identify and anticipate these scenarios in addition to early detection and prediction tactics. This assists decision makers, reduces maintenance costs, and improves operational performance. Although predictive maintenance has been around for a while, it hasn’t been widely available until recently due to the emergence of new technologies that appear to be both affordable and capable^5^. The central component of the predictive maintenance is RUL prediction. Decision-makers can use accurate and trustworthy RUL assessment results to make informed decisions about the best maintenance plans to maximize equipment utilization, prevent expensive failures, and ultimately save maintenance costs^2^. A common understanding of what constitutes an unusual instance is lacking in anomaly detection research. Several paradigms for the creation and testing of algorithms arise from differences in the nature of an anomaly itself. An exception to this rule is predictive maintenance, in which the anomaly signifies a breakdown that needs to be stopped^6^. DL is being used more and more throughout the CPSs development lifecycle. Critical applications for these CPSs include medical infrastructure, robotics, autonomous vehicles, smart power grids, and water treatment and distribution networks^7^. Both CPSs and DLs have inherent uncertainty that, if not appropriately addressed, may cause CPSs to behave in an unreliable manner, such as an unsafe manner. The community has a good understanding of this problem. Many DL solutions for CPS data only generate labels for the input data without quantifying the uncertainty in their predictions; this can further engender mistrust in these solutions and result in unsafe and unpredictable CPS behaviors^8^. As a result, when using DL models for CPS data, a framework for quantifying uncertainty in the models must be included. This is followed by the development of mechanisms to address the uncertainty in the models. Because deep neural networks contain multilayered nonlinear designs, which have been criticized for being opaque and having predictions that are hard for humans to understand, they are considered “black box” models. Such black-box machine learning methods have generated crucial predictions in the field of CPS^9^. ML approaches have been used in Predictive Maintenance on a few prior occasions, including for rotary machine fault diagnosis and sensor failure detection in aero-engine control systems^10^. The use of ML approaches in Predictive Maintenance systems presents a number of issues, one of which is the lack of sufficient data to build a prediction model. For these types of health monitoring tasks, the anomaly detection approach^11^ is consequently more suitable since it can detect machine issues based on departures from healthy data^4,12,13^. This study focuses on utilizing optimized deep learning approaches to improve anomaly identification in predictive maintenance for cyber-physical systems, tackling significant problems such as data heterogeneity, limited labeled abnormalities, and model uncertainty. The impetus arises from the necessity to mitigate unanticipated downtime, lower maintenance expenditures, and enhance operational efficiency in intricate industrial settings, where conventional maintenance approaches are inadequate due to their reactive characteristics or dependence on predetermined intervals that fail to consider real-time system health. The primary research question of this project is: How can the accuracy of anomaly identification in predictive maintenance for cyber-physical systems be enhanced by integrating trend factor smoothing with bio-inspired optimization and siamese neural networks? This study seeks to implement optimum deep learning methodologies for an enhanced model of anomaly detection. The suggested methodology consists of feature selection, anomaly injection, anomaly detection, and pre-processing phases. Initially, the box-cox transformation is utilized for the pre-processing of input data^14^. Subsequently, TFS^15^ is included into TDO^16^ to introduce an innovative method, termed TFsTDO, aimed at optimizing the feature selection process by selecting relevant features effectively. Anomalies for predictive maintenance are ultimately identified using the SNN methodology, which is taught by the proposed TFsTDO algorithm. The subsequent delineates the major contributions of the mechanism:

Developing a unique approach, termed TFsTDO, that incorporates Trend Factor Smoothing into Tasmanian devil optimization to better feature selection and increase robustness against noise in high-dimensional CPS datasets. Utilizing the siamese neural network model for anomaly detection, trained by the proposed TFsTDO algorithm. The ability of SNN to learn from semantic similarity is one of its primary advantages, allowing it to cluster related items by understanding the spatial relationships of embeddings. Evaluating the suggested model’s efficacy through metrics such as precision, recall, and F1-score, revealing enhanced outcomes on the CNC Mill Tool Wear dataset.

This study surpasses the current state-of-the-art through the innovative incorporation of Trend Factor Smoothing (TFS) into Tasmanian devil optimization (TDO), resulting in TFsTDO, which exceeds the performance of conventional optimization techniques such as Genetic Algorithms (GA) and Particle Swarm Optimization (PSO) in managing noisy, high-dimensional data from Cyber-Physical Systems (CPS). This is evidenced by its superior exploration-exploitation balance and computational efficiency, as demonstrated on benchmark functions like Sphere and Rastrigin. In contrast to current methodologies like LSTM-autoencoders^3^, CNN-LSTM hybrids^2^, GA-based predictions^17^, or AdaBoost with random projections^18^, our TFsTDO-SNN framework not only enhances feature selection but also trains the SNN for semantic similarity detection, tackling data scarcity through anomaly injection via oversampling. This yields exceptional empirical performance (96.5% precision, 97.2% recall, 96.9% F1-score), exceeding state-of-the-art approaches by 2-5% in critical metrics, while integrating uncertainty quantification and explainability features, rendering it more appropriate for real-time cyber-physical system applications. The document is organized as depicted: Segment 1 presents an introduction. Segment 2 asserts a comprehensive examination of prior studies and research. The third segment delineates the proposed methodology. Segment 4 presents the results and discussions, while Segment 5 provides the conclusion and future scope.

## Literature survey

This section asserts earlier studies and research related to anomaly detection and predictive maintenance. Xanthi Bampoula et al.,^3^ explores a way to make it easier for people to remain from predictive maintenance duties to preventative maintenance tasks that are scheduled at predetermined intervals. In a cyber-physical production system, such techniques are made possible by a deep learning algorithm, which allows maintenance tasks to be scheduled based on the machine’s actual operational status rather than on a predetermined schedule. Real-world machine and sensor data is classified using an autoencoder-based methodology into a set of condition-related labels. Using actual data gathered from production processes, a prototype implementation of LSTM-autoencoders is trained and tested to estimate abnormalities of monitored equipment. Finally, a steel firm use case is employed to evaluate proposed methodology. Maxim Shcherbakov and Cuong Sai^2^ developed an effective hybrid deep learning multi-task framework that combines advantages of LSTM and CNN neural networks. The objective of this framework is to illustrate the relationship between health status detection and anomaly prediction in complex multi-object systems in context of CPS. Condensing condition monitoring data and immediately extracting important spatiotemporal characteristics from raw multi-sensory input data is role of CNN as a feature extractor. Conversely, extended temporal dependency aspects are captured using LSTM. The effectiveness of hybrid deep learning architecture has been confirmed via testing on well-known C-MAPSS dataset from NASA. Results show that hybrid CNN-LSTM model performs better than methods based on deep learning and standard machine learning.

**Table 1:** Summary of literature review.

Citation	Methodology	Dataset	Advantages	Key findings
Bampoula et al.^3^	LSTM-autoencoder, autoencoder-based labels	Steel firm data	Real-time scheduling, anomaly detection	Established viability of deep learning in predictive maintenance using operational status.
Shcherbakov and Sai^2^	CNN-LSTM hybrid model	NASA’s C-MAPSS dataset	High accuracy, extracts spatiotemporal features	The model efficiently links health detection and anomaly prediction in CPSs.
Basit et al.^17^	GA, neural sensing network	Textile spinning system data	Efficient, minimal training data	Showcased precision in identifying specific failures in high-speed textile systems.
Li and Niggemann^18^	AdaBoost, random projection	CPS fault diagnosis data	Handles non-convex data, flexible	Suggested a flexible and efficient approach to handle both convex and non-convex CPS fault data.
Hao et al.^19^	DRL, PPO, cyber aging models	Simulated CPS data	Decision-making under uncertainty	Enhanced cyber and hardware component regeneration techniques by integrating aging models into RL.
Dangut et al.^20^	Autoencoder, GRU, focal loss	Airplane maintenance data	Imbalance handling, effective prediction	Integration of DL with focal loss showed successful rare failure estimation in aviation.
Goetz and Humm^21^	1D convolutional autoencoders	Industrial CPS data	Decentralized, real-time processing	Examined an automated, decentralized technique for identifying anomalies in industrial CPSs.
Nagarajan et al.^22^	CNN, GMM-KF	Not mentioned	Privacy-preserving, accurate predictions	Developed a novel framework for identifying anomalies that combines CNN and GMM-KF.
Omol et al.^23^	Isolation Forest, SVM, Autoencoders	IoT smart grid data	Real-time, accurate detection	Proactive maintenance, reliability.
Kim et al.^24^	Deep learning (Autoencoders, CNN)	Smart grid data	Superior feature learning	Effective in real-world scenarios.
Martinez et al.^25^	ML-based predictive maintenance	Energy IoT data	High precision, optimization	Minimizes downtime, schedules tasks.
Chen et al.^26^	Random Forest, SVM, ensemble models	Energy system case studies	Scalable, interpretable	Enhanced anomaly detection.
Velásquez et al.^27^	Hybrid ensemble (LOF, SVM, Autoencoder)	Air-blowing machine data	Real-time, improved F1	Hybrid outperforms individual methods.
Allen et al.^28^	Knowledge-Enhanced Spatiotemporal Analysis (KESA)	Tennessee Eastman process dataset	Timely fault detection, interpretable results, cost efficiency	KESA outperforms existing algorithms in accuracy and efficiency, emphasizes domain expertise in anomaly detection.
Zhu and Shao^29^	Moath Quantile CNN, Spatial Clustering	Optoelectronic sensor data	Improved prediction accuracy, noise reduction	Effective fault detection, high precision and recall rates.
Jovanović et al.^30^	Spectrogram feature extraction classifier	Motor acoustic recordings	Non-intrusive sensing; robust feature refinement via optimization	Enhances fault representation and identification in noisy environments.
Savanović et al.^31^	Hybrid CNN–XGBoost with modified sine–cosine metaheuristic.	Network/CPS traffic benchmarks (Win7, Win10 / TON-IoT-like datasets)	Combines deep embeddings with gradient boosting; MH tuning reduces false positives.	Hybrid achieves superior detection and generalization on high-dimensional anomaly data.
Khiat et al.^32^	GA–XGBoost.	Compressor 103J and industrial water-pump datasets	Improves ensemble robustness without manual tuning.	Enhances classifier reliability on heterogeneous industrial sensor data.
Buabeng et al.^33^	CLUST–SMOTE–GWO–MLP	Hydraulic system multi-sensor dataset	Addresses class imbalance via clustered SMOTE; GWO optimizes MLP for balanced clusters.	Improves multiclass PdM consistency and mitigates imbalance effects.
Kumar et al.^34^	Metaheuristic-enhanced DL for RUL	CMAPSS / aircraft engine sensor data	Improves convergence and generalization of deep models for RUL estimation.	Yields more stable and accurate RUL predictions on aviation benchmarks.
Chen et al.^35^	Swarm-tuned XGBoost	Mining machinery time-to-failure dataset	Efficiently explore time-series feature space; robust in harsh conditions.	Improves time-to-failure forecasting reliability under noisy industrial regimes.

Farooq Basit et al.^17^ presented a GA-based prediction method for the spinning system for health assessment. Their results show that the system can operate using more data volumes while utilizing fewer training datasets. By employing intelligent agents in conjunction with the neural sensing network, this integrated system is intended to predict anomalies, disruptions, and malfunctions by means of condition-based monitoring of individual components. This approach improves the precision of identifying particular component failures within the spinning spindles. An industrial Internet of things case study was carried out to demonstrate the prediction model’s viability for high-speed textile spinning system and dynamic. Real-time data sensing and signal processing were utilized in the study. Li and Niggemann^18^ presented a non-convex version of classic one-class classification algorithm that combines AdaBoost algorithm with random projection to address non-convex data problems. This approach is appropriate for CPS analysis tasks because it has significant benefits in terms of efficiency, flexibility, and applicability to both convex and non-convex data sets. This method’s effectiveness was evaluated using CPS fault diagnosis tasks.

Hao et al.^19^ presented DRL to optimize strategies that consider the health status and RUL of system hardware components, as well as potential accident scenarios resulting from hardware and cyber component failures and aging. This research is innovative because it incorporates cyber aging model into production planning and failure processes model. This helps RL agent determine when to rejuvenate cyber system and accounts for uncertainties in cyber system aging process by training with PPO and IL. Dangut et al.^20^ established a DL method to handle extremely rare failure predictions in aviation predictive maintenance modeling, using auto-encoder and bidirectional gated recurrent unit networks. In order to forecast next failure occurrence, autoencoder’s output is fed into a convolutional bidirectional gated recurrent unit network after it has been modified and trained to identify unusual failures. Rescaled focal loss in conjunction with network architecture successfully addresses imbalance problem during model training. By contrasting it with other deep learning techniques, effectiveness of this method was assessed using real-world situations using log-based warnings and failure messages taken from airplane central maintenance system data.

Goetz and Humm^21^ developed a real-time, decentralized, and unsupervised method for identifying process abnormalities in CPSs. The technology meets real-time operating demands and ensures effective prediction accuracy by utilizing several 1D convolutional autoencoders within a sliding window structure. For enhanced adaptability and compliance with communication protocols and processing limitations commonly found in cyber-physical production setups, the execution of anomaly detection is distributed across individual cyber-physical systems. The implementation process is entirely automated, eliminating the necessity for expert input to address data-driven constraints. The efficacy of the concept is verified through assessment within an actual industrial cyber-physical production environment. The results of the evaluation validate the applicability of the presented concept in identifying anomalies across all processes within each cyber-physical system. Nagarajan et al.^22^ introduced a detection methodology that integrates CNN with KF-based GMM for anomaly detection in CPSs. The framework involves two primary stages. Initially, data preprocessing involves the transformation and filtration of raw data into a new format while ensuring data privacy. The model then employs a combination of GMM-KF and deep CNN to detect anomalies, thereby predicting posterior probabilities of both anomalous and normal occurrences in CPSs.

Recent studies, including^36^, have evidenced the efficacy of hybrid deep learning models for anomaly detection in industrial IoT settings, thereby underscoring the significance of integrating temporal and spatial feature extraction. Shitharth et al.^37^ examines intelligent intrusion detection frameworks utilizing deep neural architectures, emphasizing the increasing significance of anomaly detection models in cybersecurity-oriented predictive maintenance systems. Likewise, Prashanth et al.^38^ presents a comparative review of contemporary IDS models, highlighting the benefits of hybrid anomaly detection methods that optimize efficiency and detection precision. Furthermore, Selvarajan et al.^39^ underscores the significance of optimization-based anomaly detection methodologies, reinforcing our strategy of using TFsTDO to enhance feature selection and predictive maintenance efficacy. Moreover, Shitharth et al.^40^ illustrates the efficacy of deep learning-based intrusion detection frameworks, highlighting its relevance in real-time anomaly detection inside cyber-physical systems.

Lately, tremendous research work has been carried out on hybrid approaches that integrate metaheuristic optimization with machine-learning models to tackle issues in predictive maintenance, including noisy, high-dimensional sensor data, few labeled anomalies, and model interpretability. In such combinations, metaheuristics function as global optimizers-choosing features, adjusting hyperparameters, or modifying model architectures-while the machine learning component performs fault categorization, predictive modeling, or anomaly detection.

Jovanović et al.^30^ introduced an Acoustic-based predictive maintenance methodology for manufacturing machinery, utilizing an improved metaheuristic algorithm to enhance acoustic feature selection and classification features. Their approach utilized features extracted from spectrograms of motor data, while the genetic optimizer continually enhanced feature subsets and model hyperparameters to improve fault-detection accuracy. This non-intrusive sensing approach demonstrated significant performance enhancements compared to traditional classification standards, highlighting the ability of metaheuristics to identify meaningful representations despite limited labeled data. The research demonstrated that optimization-driven feature engineering can markedly improve the diagnostic accuracy of predictive maintenance systems functioning amidst real-world noise and ambiguity.

Savanović et al.^31^ investigated a hybrid CNN–XGBoost framework optimized by a modified sine–cosine algorithm for intrusion detection and cloud security, a field closely related to CPS anomaly identification. Their hybrid model employed CNNs to derive discriminative embeddings from raw network data, while XGBoost managed tabular classification; the metaheuristic concurrently improved hyperparameters and feature selections, resulting in enhanced precision in detection and diminished false-positive rates relative to untuned or traditionally tuned baselines. The findings highlighted the efficacy of metaheuristic search in optimizing hybrid deep-ensemble models for intricate, high-dimensional data settings.

Khiat et al.^32^ introduced a hybrid framework that combines the Genetic Algorithm (GA) with Extreme Gradient Boosting (XGBoost) for predictive maintenance of industrial equipment, including compressors and water pumps. Their methodology utilizes the global optimization capabilities of GA to optimize the hyperparameters of XGBoost, facilitating more accurate categorization of machine states amongst heterogeneous and noisy sensor data. The research illustrates how evolutionary optimization may proficiently navigate the high-dimensional parameter space of ensemble models, reducing overfitting and enhancing generalization without requiring lengthy human calibration. The authors demonstrate a viable approach to improving diagnostic accuracy in industrial maintenance by integrating metaheuristic search with gradient boosting, ensuring computational efficiency and model interpretability.

Buabeng et al.^33^ introduced a hybrid clustering–SMOTE–MLP model optimized with the Grey Wolf Optimizer (GWO) to address multiclass and imbalanced fault scenarios in hydraulic systems. Their methodology revealed that integrating unsupervised clustering with metaheuristically optimized neural networks enhances classification consistency and alleviates data imbalance issues. Kumar et al.^34^ devised a metaheuristic-enhanced deep learning framework for predictive maintenance of aircraft engines, utilizing optimization algorithms to refine neural network hyperparameters for remaining useful life (RUL) prediction, thereby effectively tackling high-dimensionality and non-linearity in aviation sensor data. In a separate study, Chen et al.^35^ utilized XGBoost-based metaheuristic techniques to predict time-to-failure in mining machinery, demonstrating that the incorporation of swarm-inspired optimization into ensemble learning can markedly enhance the reliability of time-series forecasting in challenging industrial environments. In addition to these initiatives, El-Ariss et al.^41^ evaluated various hybrid machine learning methodologies for predictive maintenance jobs and emphasized that metaheuristic optimization of model architectures consistently produces superior predictive performance compared to traditional machine learning pipelines.

Based on the key findings with current methodologies mentioned in the previous section and in Table 1, the fact that though the details on the machine’s health is provided in many works without necessitating any further training or specialized from industry operators, it is noted that there is a major scope in improving the study by focusing on tuning and optimizing parameters as well as using advanced ML techniques. Some frameworks mentioned in the study offer significant assistance for the creation of maintenance plans and health management for intricate multi-object systems; although there is room for improvement, the models have established a strong basis on which optimization can be applied. Analysis of the existing state-of-the-art reveals that optimization-driven methodologies provide significant potential to enhance classifier efficacy and predictive accuracy. The current study introduces the trend factor smoothing-Tasmanian devil optimized siamese neural network (TFsTDO-SNN), which integrates a trend-aware metaheuristic into both the feature-selection and model-training stages. This co-optimized deep learning approach seeks to strengthen dynamic robustness, promote interpretability, and provide more precise and adaptable predictive maintenance for intricate cyber-physical systems.

### Rationale for using Tasmanian devil optimization (TDO)

The choice of Tasmanian devil optimization (TDO) as the fundamental algorithm for the proposed TFsTDO framework is motivated by its bio-inspired metaheuristic characteristics, which are particularly effective for feature selection in predictive maintenance of cyber-physical systems (CPS). TDO, introduced by Dehghani et al.^42^, emulates the foraging behavior of Tasmanian devils, utilizing dual techniques of *exploration* (finding carrion) and *exploitation* (pursuing prey) to traverse intricate search spaces. This equilibrium renders TDO efficacious for high-dimensional challenges, such as identifying pertinent characteristics from the noisy CNC Mill Tool Wear dataset^43^, which comprises sensor data including vibration signals and spindle load.

TDO’s resilience to noise, computational efficiency, and equilibrium between exploration and exploitation set it apart from alternatives such as Genetic Algorithms (GA) and Particle Swarm Optimization (PSO). Genetic algorithms, although effective for global optimization, are computationally demanding because of crossover and mutation processes, rendering them less appropriate for real-time applications^44^. Particle Swarm Optimization (PSO) facilitates rapid convergence; yet, it is susceptible to premature convergence in multimodal landscapes owing to its sensitivity to parameters^44^. TDO’s streamlined update rules (Eqs. 4–7) and adaptive search method offer a robust and efficient alternative, as demonstrated by its performance on benchmark problems such as Sphere and Rastrigin^45^.

Selvarajan^46^ emphasizes the effectiveness of bio-inspired metaheuristics, such as TDO, in engineering applications, highlighting their simplicity, adaptability, and capacity to address difficult issues like feature selection and fault diagnostics. The comparative analysis in this article emphasizes techniques such as Horse Herd Optimization (HHO), which exhibit similar resilience and rapid convergence as TDO, hence affirming TDO’s appropriateness for CPS datasets. The anticipated advantages of TDO encompass improved feature selection precision, which aids in achieving the SNN’s elevated recall (97.2%) and F1-score (96.9%), as well as diminished computing burden, essential for real-time predictive maintenance.

The incorporation of Trend Factor Smoothing (TFS) in TFsTDO further alleviates scaling issues identified by Selvarajan^46^, improving efficiency by refining noisy time-series data. The aforementioned attributes, coupled with TDO’s historical efficacy in optimization endeavors^45^, substantiate its preference over GA and PSO, in accordance with the review’s focus on bio-inspired methodologies for engineering problems.

## Proposed methodology: TFsTDO-SNN based anomaly detection for predictive maintenance

**Figure 1:**
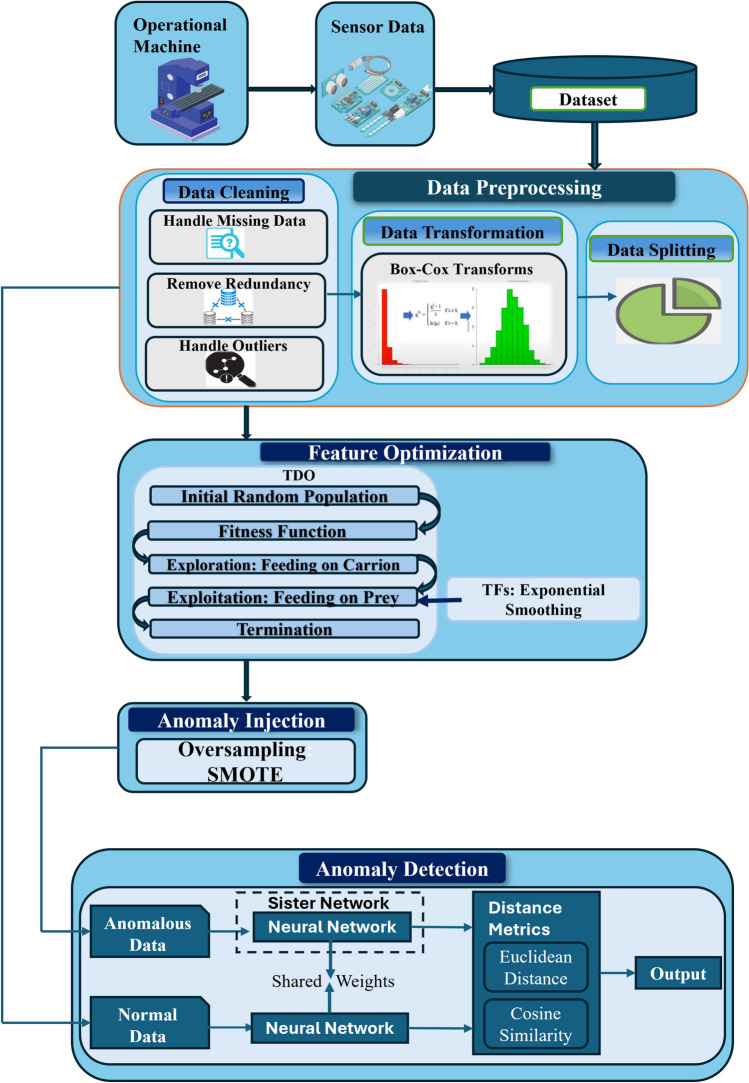
Architecture diagram of presented anomaly detection and predictive maintenance.

This work attempts to use an enhanced model for anomaly identification using deep learning technique. The proposed approach includes several stages, such as pre-processing, feature selection, anomaly injection and anomaly detection, as stated in Fig. 1. Method of operating machinery to prevent the occurrence of faults or to anticipate their occurrence at specific times and locations represents the prospective focus of the field of equipment operation and maintenance.

The initial dataset is characterized by its heterogeneity, size, variety of classes, and dynamic nature, necessitating the preprocessing of a substantial volume of unprocessed data. Utilizing a Deep Learning approach is crucial for analysis and computation of the data, which involves identifying the key data features and examining the principal components of these features. Here, box-cox transformation is used to accomplish pre-processing^14^. Following that, the TFsTDO-based feature selection process is carried out through the integration of TFS^15^ in TDO^16^ to increase the models’ accuracy and resilience by choosing the most informative features. The benefits of TFS and TDO combined can produce a robust and effective feature selection process. Next, the SNN model^47^, which has been trained using the suggested TFsTDO, is used to detect anomalies. SNNs are made to compare pairs of input samples in order to identify patterns in the data. Because they can discriminate between normal and abnormal patterns, they become more resilient to outliers and anomalies. By adopting this approach, it becomes feasible to implement proactive maintenance measures to prevent malfunctions, thereby ensuring uninterrupted production, product excellence, and operational efficiency.

### Preprocessing

Preprocessing is the process of transforming unstructured, noisy, and raw data into a format that can be analyzed. Here, the Box-Cox transformation is used to carry out the preprocessing step.

The Box–Cox power transformation^14^, first introduced by Box and Cox in 1964, is used to stabilize variance, eliminate skewness, and transform time series into distributions that are more akin to normal. Assuming that the variable $A_l(l=1,2,\ldots n)$ is a positive random variable, the definition of the Box-Cox transformation with parameter $\beta$ is:

1 $$\begin{aligned} A_l^\beta = {\left\{ \begin{array}{ll} \frac{A_l^\beta - 1}{\beta }, & \beta \ne 0 \\ \log (A_l), & \beta = 0 \end{array}\right. }, \quad l = 1, 2, \ldots , n \end{aligned}$$

where $A_l$ represents the original data ($A_l> 0$), $A_l^\beta$ denotes the transformed data, and $\beta$ is the transform coefficient.

### TFsTDO based feature selection

The TFsTDO algorithm represents a new technique for feature selection that amalgamates the capabilities of TFS^15^ and TDO^16^ in order to pinpoint the most pertinent features, thereby enhancing the accuracy of feature selection. This heuristic algorithm systematically seeks the optimal solution by choosing features that exhibit high relevance to target variable in an iterative manner, while simultaneously excluding features with low relevance. TFS, which is a forecasting technique, plays a crucial role in forecasting with the smoothing factor so as to fit the data based on user’s preferences. Thus, the incorporation of TFS in TDO helps in selecting the most suitable features so as to assist the classifier in improving the detection performance. Moreover, this algorithm demonstrates computational efficiency, particularly in contrast to alternative feature selection approaches that demand substantial computational resources.

#### Solution encoding

**Figure 2:**
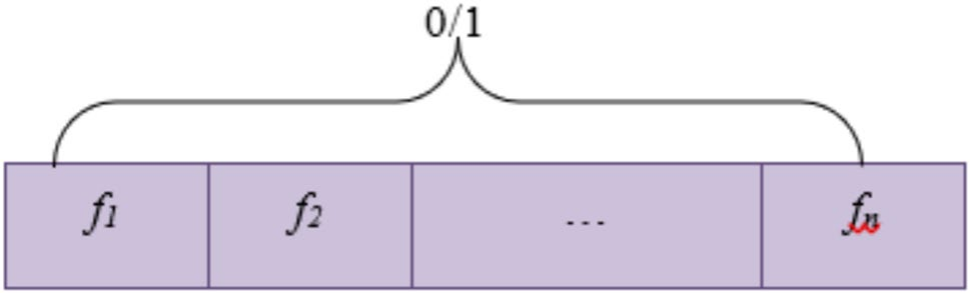
Solution encoding.

The number of variables or decision-making components that are simultaneously improved for specific problems in optimization to produce better outcomes is called the “solution dimension.” The characteristics of this are represented by solution size 1$\times$n, which might have values of zero or one. Feature location value of one is chosen in this instance. Figure 2 shows TFsTDO’s solution encoding.

#### Tasmanian devil optimization trend factor

TDO is a bio-inspired metaheuristic algorithm designed by mimicking natural behavior of Tasmanian devils. Tasmanian devil, which has two feeding strategies attacking live prey or consuming carrion of deceased animals, is main source of inspiration for TDO. Optimization findings show how proficient TDO is at both exploration and exploitation, and they strike right balance between these two metrics to address optimization issues with efficiency. However, use of TDO in finding important features for this topic has to be explored by modifying it with integration of TFS, which is named TFsTDO. Steps in the proposed TFsTDO are explained below:

- *Step 1: Initialization*
  Tasmanian devil optimization (TDO) is a population-based stochastic algorithm, in which Tasmanian devils are search agents. A random selection is made for the initial population of these agents in order to account for issue constraints. TDO allows a set of population members known as “explorers” to provide potential solutions by virtue of their presence inside the search region. These explorers offer suggestions for choice variables, which sets off an inquiry into the field of problem solving. Because of this, each person in the population can be viewed as a vector whose components all match the number of variables in the problem. As a result, Eq. (2) can be used to represent a group of members as a matrix:
  2 $$\begin{aligned} P = \begin{bmatrix} P_1 \\ \vdots \\ P_v \\ \vdots \\ P_N \end{bmatrix}_{N \times M} = \begin{bmatrix} p_{1,1} & \cdots & p_{1,r} & \cdots & p_{1,M} \\ \vdots & \ddots & \vdots & \ddots & \vdots \\ p_{v,1} & \cdots & p_{v,r} & \cdots & p_{v,M} \\ \vdots & \ddots & \vdots & \ddots & \vdots \\ p_{N,1} & \cdots & p_{N,r} & \cdots & p_{N,M} \end{bmatrix}_{N \times M} \end{aligned}$$
  where $P$ is the Tasmanian devils’ population, $N$ is the number of Tasmanian devils in the search, $M$ is the number of variables present in the given problem, $P_v$ is the $v$th candidate solution, and $p_{v,r}$ is its value for the $r$th variable.
- *Step 2: Fitness*
  Fitness is a measurement of how well a model works on a particular task or issue. It is frequently used to compare an algorithm’s or model’s performance with that of other models or algorithms, as well as to assess how well it performs on a specific dataset. Here, recall of the transformed data measured using a neural network is considered as fitness. Hence, solution having maximum fitness, i.e. recall, is considered as the optimal solution.
- *Step 3: Feeding by Eating Carrion (Exploration Phase)*
  Tasmanian devil may occasionally choose to eat local carrion rather than go hunting. Tasmanian devil shares its environment with other predators that hunt larger prey but have difficulty finishing it off. The strategies Tasmanian devils use to find carrion in their surroundings is similar to the strategy algorithms used to traverse problem domains. This specific tactic demonstrates how well Tasmanian devil exploration works to identify the best starting point by scanning various areas of search area. By utilizing Eqs. (3) to (5), a mathematical representation of concepts outlined in Tasmanian devil’s carcass consumption strategy is formulated.
  3 $$\begin{aligned} C_v = P_q, \quad v = 1, 2, \ldots , N, \quad q \in \{1, 2, \ldots , N \mid q \ne v\} \end{aligned}$$
  where $C_v$ is the carrion selected by the $v$th Tasmanian devil, and $q$ is a random number from one to $N$.
  4 $$\begin{aligned} & p_{(v,r)}^{(t+1, w_1)} = {\left\{ \begin{array}{ll} p_{(v,r)}^t + u \cdot (b_{(v,r)} - c \cdot p_{(v,r)}^t), & F_{C_v}^t < F_v^t, \\ p_{(v,r)}^t + u \cdot (b_{(v,r)} - p_{(v,r)}^t), & \text {otherwise}. \end{array}\right. } \end{aligned}$$
  5 $$\begin{aligned} & P_v = {\left\{ \begin{array}{ll} P_v^{(t+1, w_1)}, & F_v^{(t+1, w_1)} < F_v^t, \\ P_v^t, & \text {otherwise}. \end{array}\right. } \end{aligned}$$
  wherein, $P_v^{(t+1, w_1)}$ is based on the first strategy of the Tasmanian devil’s new status of the *v*th variable. The term $p_{(v,r)}^{(t+1, w_1)}$ is the *r*th variable value. $F_v^{(t+1, w_1)}$ denotes the value of the objective function, while $F_{C_v}^t$ is the selected carrion objective function value. The variable *r* is a random number in the interval [0, 1], and *c* is a random count that can be either one or two. Finally, $F_v^t$ represents the *v*th candidate solution of the objective function value.
- *Step 4: Feeding by Eating Prey (Exploitation Phase)* The Tasmanian devil’s secondary means of livelihood is hunting and feeding. The location of Tasmanian devil is updated as soon as prey’s whereabouts are verified. If the selected prey’s objective function value is higher, it travels to this updated position; if not, it departs from that location. Equation (6) provides an illustration of this procedure’s representation. If Tasmanian devil’s new position increases the value of target function, it replaces old one. Equation (7) illustrates the second approach’s evolution.
  6 $$\begin{aligned} & p_{(v,r)}^{(t+1, w_2)} = {\left\{ \begin{array}{ll} p_{(v,r)}^t + u \cdot (b_{(v,r)}^t - c \cdot p_{(v,r)}^t), & F_{j_v}^t < F_v^t, \\ p_{(v,r)}^t + u \cdot (p_{(v,r)}^t - b_{(v,r)}^t), & \text {otherwise}. \end{array}\right. } \end{aligned}$$
  7 $$\begin{aligned} & P_v^t = {\left\{ \begin{array}{ll} P_v^{(t+1, w_2)}, & F_i^{(t+1, w_2)} < F_v^t, \\ P_v^t, & \text {otherwise}. \end{array}\right. } \end{aligned}$$
  here $P_v^{(t+1, w_2)}$ is based on second strategy Tasmanian new status of *v*th, $p_{(v,r)}^{(t+1, w2)}$ is *r*th variable value, $F_i^{(t+1,w2)}$ denotes value of objective function, and $F_{(j_v)}^t$ is the objective function value of selected prey.
  At this instance, Tasmanian devil’s whereabouts is thought to be middle of area where the hunt is taking place. Boundaries of this area, which may be computed using Eq. (8), indicate range throughout which Tasmanian devil pursues its victim. Tasmanian devil will accept newly calculated position if it produces a higher value for objective function than previous one.
  8 $$\begin{aligned} R_d = 0.01 \cdot \left( 1 - \frac{G}{g} \right) \end{aligned}$$
  wherein,*Rd* is the neighborhood radius of the point of attacked location, *G* is iteration counter, and *g* is maximum iterations. For Tasmanian devil, new position that arises from pursuing procedure in this area may be calculated, and this is mathematically repeated in Eq. (9).
  9 $$\begin{aligned} & P_{(v,r)}^{(t+1)} = P_{(v,r)}^t + (2y - 1) \cdot R_d \cdot P_{(v,r)}^t \end{aligned}$$
  10 $$\begin{aligned} & P_{(v,r)}^{(t+1)} = P_{(v,r)}^t \left[ 1 + (2y - 1) \cdot R_d \right] \end{aligned}$$
  $P_{(v,r)}^{(t+1)}$ is *r*th variable value of new Tasmanian devil in neighborhood.
  TFS is combined with TDO to get global optimal solution. Using TFS modifies Tasmanian devil’s position within search space.
  11 $$\begin{aligned} P^t = \left( X^{(t-1)} + Y^{(t-1)} \right) \cdot S^{(t-x)} \end{aligned}$$
  Assuming, $$x = 1$$,
  12 $$\begin{aligned} P^t = \left( X^{(t-1)} + Y^{(t-1)} \right) \cdot S^{(t-1)} \end{aligned}$$
  where
  13 $$\begin{aligned} & X^{(t-1)} = \nu \cdot \frac{e^{(t-1)}}{S^{(t-2)}} + (1 - \nu ) \cdot \left( X^{(t-2)} + Y^{(t-2)} \right) \end{aligned}$$
  14 $$\begin{aligned} & Y^{(t-1)} = \gamma \cdot \left( S^{(t-1)} - S^{(t-2)} \right) + (1 - \gamma ) \cdot Y^{(t-2)} \end{aligned}$$
  15 $$\begin{aligned} & S^{(t-1)} = \epsilon \cdot \frac{e^{(t-1)}}{S^{(t-1)}} + (1 - \epsilon ) \cdot S^{(t-2)} \end{aligned}$$
  where $\nu$, $\gamma$, and $\epsilon$ smoothing constant in which $0< \nu < 1$, $0< \gamma < 1$, and $0< \epsilon < 1$, respectively. Substituting the values of $X^{(t-1)}$, $Y^{(t-1)}$, and $S^{(t-1)}$, in Eq. (12),
  16 $$\begin{aligned} \begin{aligned} P^t&= \left[ \nu \cdot \frac{e^{(t-1)}}{S^{(t-2)}} + (1 - \nu ) \cdot \left( X^{(t-2)} + Y^{(t-2)} \right) \right. \\&\quad + \gamma \cdot \left( S^{(t-1)} - S^{(t-2)} \right) + \left. (1 - \gamma ) \cdot Y^{(t-2)} \right] \\&\quad \times \left[ \epsilon \cdot \frac{e^{(t-1)}}{S^{(t-1)}} + (1 - \epsilon ) \cdot S^{(t-2)} \right] \end{aligned} \end{aligned}$$
  Substituting Eq. (16) in Eq. (10), stating $P^t=P_{(v,r)}^{t}$, and update equation is defined as,
  17 $$\begin{aligned} \begin{aligned} P_{(v,r)}^{(t+1)}&= \left[ \nu \cdot \frac{e^{(t-1)}}{S^{(t-2)}} + (1 - \nu ) \cdot \left( X^{(t-2)} + Y^{(t-2)} \right) \right. \\&\quad \left. + \gamma \cdot \left( S^{(t-1)} - S^{(t-2)} \right) + (1 - \gamma ) \cdot Y^{(t-2)} \right] . \end{aligned} \end{aligned}$$
  where $\nu$, $\gamma$, and $\epsilon$ smoothing constant in which $0< \nu < 1$, $0< \gamma < 1$, and $0< \epsilon < 1$, respectively. $X^t$ is de-seasonalized level estimate, $Y^t$ is trend estimate, $S^t$ is seasonal component estimate , *r* is random number (0,1) and *Rd* is radius of neighborhood.
- *Step 5: Termination* Above steps are taken until the maximum number of iterations is reached, at which time the optimization process produces the optimal result. Algorithm 1 presents the pseudocode for TFsTDO.

**Algorithm 1:**
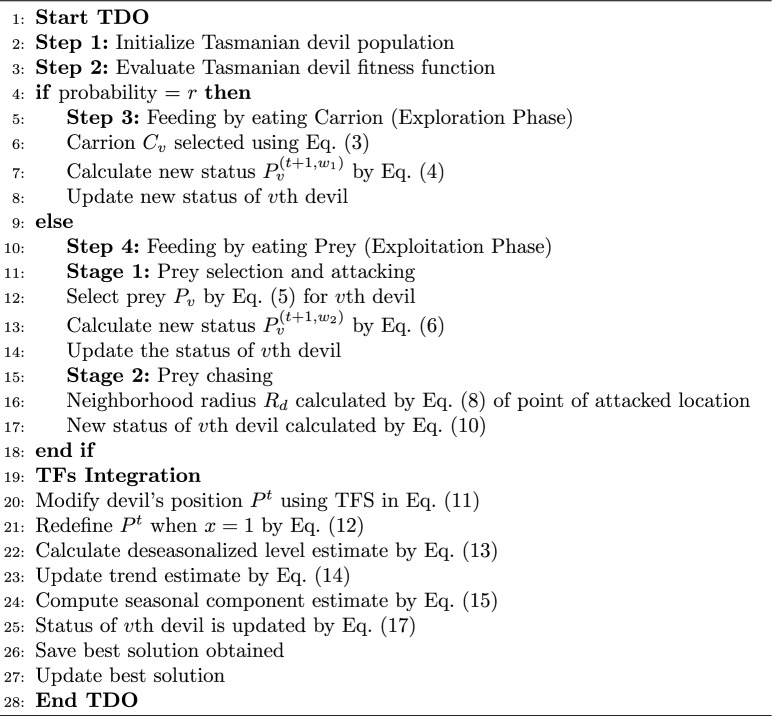
Pseudocode of TFsTDO

The mathematical modeling in the proposed TFsTDO-SNN framework is essential for optimizing feature selection and improving anomaly identification. The TFsTDO equations delineate the adaptive exploration–exploitation method, wherein each iteration refines potential solutions based on fitness values obtained from feature relevance. The optimized features are then utilized by the Siamese Neural Network (SNN), which employs similarity metrics and contrastive loss functions to differentiate between normal and abnormal patterns. The integration of optimization results with the anomaly detection model directly enhances the accuracy of parametric evaluations, such as precision, recall, and F1-score.

**Table:** 

Symbol	Description
*P*	Population matrix of Tasmanian devils
*N*	Number of Tasmanian devils (population size)
*M*	Number of variables/features to optimize
$P_v$	$v^{th}$ candidate solution in the population
$p_{v,r}$	Value of the $r^{th}$ variable for the $v^{th}$ solution
$C_v$	Carrion selected by the $v^{th}$ devil
$F_v$	Fitness value of the $v^{th}$ candidate
*b*(*v*, *r*)	Best position of the $v^{th}$ variable
*u*	Random step size factor
*c*	Strategy selection constant
*Rd*	Neighborhood radius
*G*	Current iteration count
*g*	Maximum number of iterations
*X*(*t*)	De-seasonalized level estimate (TFS)
*Y*(*t*)	Trend estimate (TFS)
*S*(*t*)	Seasonal component estimate (TFS)
$\nu , \gamma , \epsilon$	Smoothing constants in TFS
*K*	Class label indicator (1 = same-class, 0 = different-class)
$K_{pred}$	Predicted label from SNN
*L*	Euclidean distance between SNN outputs
$\omega$	Margin threshold in contrastive loss

### Oversampling data by anomaly injection

The process of oversampling data involves generating anomalous data by exceeding the bound values of the original data. Oversampling data through anomaly detection entails the identification and integration of data points to enhance the efficacy of machine learning models. Algorithms designed for anomaly detection are capable of pinpointing these atypical data points, which often signify crucial patterns or relationships that might escape detection within conventional data sets. Through the incorporation of these anomalies into the dataset, models can acquire the ability to detect and accommodate these uncommon patterns, thereby resulting in enhanced precision and resilience. This methodology proves especially beneficial in scenarios where the scarcity of specific data points holds significance for the problem at hand, such as in fault detection. Moreover, oversampling anomalies can serve to mitigate the repercussions of class imbalance issues, wherein one class exhibits a considerably larger number of occurrences compared to others, by artificially inflating the instances within the minority class.

### TFsTDO-SNN based anomaly detection

**Figure 3:**
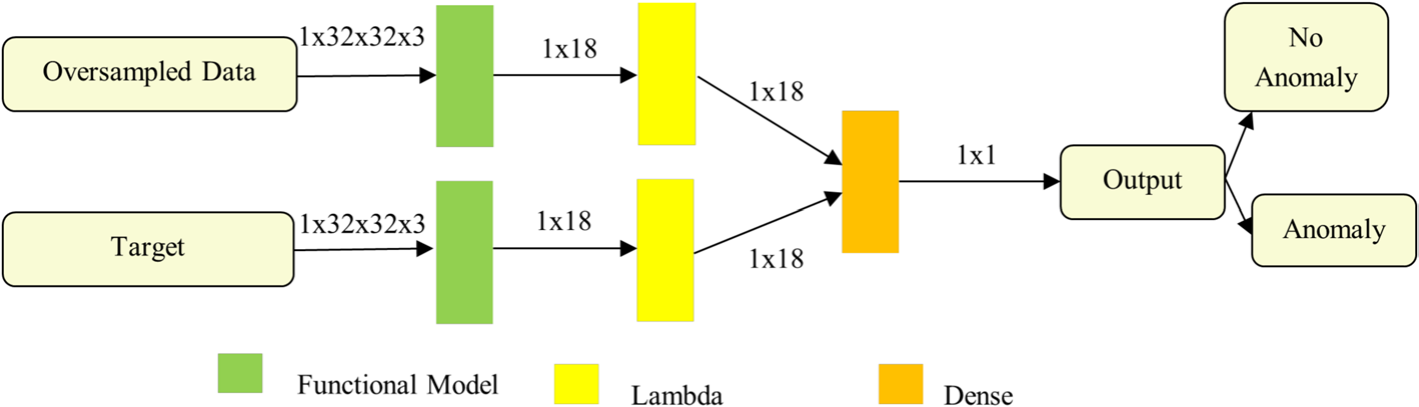
TFsTDO-SNN based anomaly detection.

The SNN^47^ is an ANN design composed of many identical FNN linked at the output and sharing weights. One element for each network is processed simultaneously in order to compare them. Lastly, a distance metric, like the Euclidean distance, is used to compare the outputs and determine whether the values differ or are comparable. Here the SNN is trained using TFsTDO. The oversampled data and anomaly target is given to TFsTDO-SNN. The output can be determined as no anomaly and anomaly. Using a loss function, this outcome is compared to the data labeling during training to assess the model’s effectiveness. The utilization of TFsTDO in training the SNN for anomaly detection presents a compelling fusion that confers numerous benefits. The TFsTDO based anomaly detection is stated in Fig. 3. Through the application of the TFS methodology, the network can diminish noise and apprehend fundamental patterns within the data, thereby enhancing its anomaly detection accuracy. The SNN empowers the network to grasp intricate associations among diverse time series data, rendering it suitable for various applications. Moreover, the TFsTDO algorithm effectively navigates the extensive hyperparameter space to ascertain the optimal network configuration, ensuring robust regularization of the model and its adeptness in generalizing to novel data. The inception of the SNN algorithm was initially presented by Bromley et al.^48^ for the purpose of detecting counterfeit signatures. ANN is a multilayer neural network model that is inspired by biological neurons, which serve as the basic processing units. Conventional supervised neural networks, also called FNN, are built using the perceptron model. A genuine input value is ingested by every neuron in the first layer of a FNN, multiplied by a weight, and then sent to every neuron in the second layer. The leftmost layer is referred to as first layer and rightmost layer as output layer in most neural network representations. The neurons in each layer after the input layer perform similar functions and communicate the results to the layer after that, ending at the top layer. The final layer of a supervised neural network then sends its outputs to a single-neuron output layer, which produces the neural network’s real-valued output.

During the training process, neural network computes statistical error by comparing the values it generates with the corresponding ground truth. Afterwards, the neural network uses a technique known as error back-propagation to feed the error back to the layers that came before it and adjusts the neuron weights accordingly. Representation-wise, back-propagation proceeds from left to right. The training culminates upon the neural network attaining the pre-established maximum number of iterations. Once trained, the model can be applied to the test set, where the neural network creates a prediction by sequentially processing each occurrence of test data. Once a predicted value has been generated for each test set instance, the programmer can use that value to create a confusion matrix. It is advised to use the MCC or the PR curve rather than possibly deceptive metrics like accuracy, F1 score, or ROC curve when evaluating a confusion matrix. Within SNNs, FNN with error back-propagation is also used. SNN’s architecture consists of two identical FNN that are connected at their output and run simultaneously. Every neural network has a standard perceptron model installed. Each neural network analyzes a profile with actual values during training and computes these values at each layer. Based on these values, the neural network activates particular neurons, uses error back-propagation to modify its weights, and finally generates an output profile that is contrasted with the output of the other neural network. The approach uses a distance metric in the original model, the cosine distance-to evaluate the output of the upper and lower neural networks. The neural network determines whether the two profiles are similar or dissimilar using this similarity assessment. The program then determines whether the data instance is positive in the first situation or negative in the second. The final output value can then be compared to the associated ground truth value, and a confusion matrix can be created by using all of the category outputs.

SNNs are sometimes called Twin Neural Networks since they accept two elements as input. The technique uses a loss function to assess the similarity between these paired items. Contrastive loss and Binary Cross Entropy are two common loss functions used in these models. Binary Cross Entropy function specifically determines if the two items are members of the same class or distinct classes.

18 $$\begin{aligned} \text {Loss} = K \cdot \left( -\log (K_{\text {pred}}) \right) + (1 - K) \cdot \left( -\log (1 - K_{\text {pred}}) \right) \end{aligned}$$

where *K* is label value. It will be one if both pairs belong to same class and zero otherwise, and $K_pred$ are label value predicted by Siamese network. Conversely, since goal of Siamese Networks is to distinguish between two elements,

Contrastive loss is a loss function that is more appropriate for task at hand.

19 $$\begin{aligned} \text {Loss} = K \cdot L^2 + (1 - K) \cdot \max \left( \omega - L, 0 \right) ^2 \end{aligned}$$

where *L* is Siamese network’s Eucledian Distance between output of its two sister networks. $\omega$ is margin, a minimal separation that attempts to distinguish between close and distant samples. It has a default value of 1.

In contrast to traditional optimizers such as GA, PSO, and CNN-LSTM, the proposed TFsTDO-SNN framework achieves a superior equilibrium between exploration and exploitation, leading to expedited convergence and enhanced feature relevance with the incorporation of Trend Factor Smoothing (TFS). These enhancements improve the accuracy and resilience of anomaly identification relative to current state-of-the-art methods, as summarized in Table 2. It offers a comparative analysis of optimization strategies, feature selection efficacy, convergence rate, detection precision, and constraints among existing methodologies in relation to the suggested framework.

**Table 2:** Comparative summary of TFsTDO-SNN versus existing methods.

Method	Optimization strategy	Feature selection	Convergence speed	Detection accuracy	Key limitation
GA	Evolutionary, random search	Limited	Moderate	Moderate	Prone to local minima
PSO	Swarm-based, velocity updates	Partial	Moderate	Good	Sensitive to parameter tuning
CNN-LSTM	Deep learning, hybrid feature extraction	Implicit	High computational cost	High	Requires large datasets
Autoencoders	Reconstruction-based anomaly detection	Implicit	Moderate	High for balanced data	Performance drops on imbalance
Proposed TFsTDO-SNN	Hybrid TDO + Trend Factor Smoothing + SNN	Explicit, optimized	Fast	Highest	Requires optimization tuning

### Overfitting mitigation

To enhance the resilience and generalization of the TFsTDO-SNN model, various approaches were utilized to alleviate overfitting, a significant issue when training deep learning models on intricate datasets like the CNC Mill Tool Wear dataset^49^. These tactics encompass L2 regularization, 5-fold cross-validation, and dropout layers, all aimed at improving model efficacy on novel data.

*L2 Regularization* L2 regularization was implemented on the network weights, with $\lambda = 0.01$, imposing a penalty on substantial weight magnitudes. This constraint diminishes model complexity, promoting smoother representations and decreasing the danger of overfitting.

*5-Fold cross-validation* The dataset was partitioned into five equal segments to evaluate stability. Each fold served once as validation, while the other four were utilized as training data. This facilitated comprehensive assessment across several data partitions and mitigated selection bias.

*Dropout layers:* A dropout rate of 0.3 was implemented in the fully connected layers, randomly disabling neurons during training. This inhibits co-adaptation and enhances generalization.

## Results

The proposed framework is implemented using Python tool, and the proposed algorithm’s performance is analyzed using evaluation criteria like F1-score, precision, and recall. The CNC Mill Tool Wear dataset^49^ is utilized for the experimentation.

### Dataset description

CNC Mill Tool Wear dataset^49^ is an essential source of information for tracking tool wear in CNC milling operations. The detection of tool wear is essential for efficient manufacturing since it affects both product quality and operational effectiveness. This dataset consists of sensor readings taken during machining operations, such as vibration signals, cutting force, and spindle load. It can be used in supervised learning applications with labeled data that represents different wear conditions, allowing users to create predictive models for real-time tool wear detection. Researchers and practitioners can use the dataset to assess performance metrics, investigate machine learning techniques, and improve maintenance plans in industrial environments. Users can gain a better understanding of the factors influencing tool wear by utilizing this data, which will ultimately increase productivity and decrease downtime in CNC milling operations.

### Experimental setup

The TFsTDO-SNN framework was developed in Python 3.11 with TensorFlow 2.11.0 and Keras for deep learning. The libraries utilized were NumPy 1.21.6, Pandas 1.3.5, scikit-learn 1.0.2, SciPy 1.7.3, Matplotlib 3.5.3, and imbalanced-learn 0.10.1 (SMOTE). Experiments were performed on a machine equipped with an Intel Core i7 (12th Gen) processor operating at 3.2 GHz, 16 GB of RAM, and an NVIDIA GTX 1660 Ti GPU, running Windows 11. All tests were conducted in an identical computing environment to guarantee reproducibility.

### Performance metrics

Section states metrics used to evaluate performance of presented TFsTDO-SNN based anomaly detection. The efficacy of the proposed TFsTDO-SNN framework is assessed by accuracy, precision, recall, and F1-score, given its significance in anomaly detection and predictive maintenance. Among these, recall is especially vital as it assesses the capacity to accurately detect abnormalities, therefore reducing the likelihood of overlooked flaws that may result in unforeseen failures. Precision is crucial for minimizing false alarms, ensuring that maintenance activities are initiated solely when warranted. The F1-score offers a comprehensive perspective by integrating precision and recall, rendering it appropriate for addressing class imbalance frequently encountered in fault detection datasets. Accuracy provides a comprehensive assessment of proper classifications but may be less dependable in significantly skewed situations. These measures collectively offer a thorough assessment, emphasizing the trade-offs between reducing false positives and false negatives.

#### Recall

The recall metric, often called sensitivity, measures proportion of real positive samples that the model properly detects.

20 $$\begin{aligned} \text {Recall} = \frac{tp}{tp + fn} \end{aligned}$$

where $tp$ is true positive, $fn$ is false negative.

#### Precision

Precision assesses proportion of projected positive instances that are actually positive. It is often referred to as positive predictive value or precision-recall trade-off.

21 $$\begin{aligned} \text {Precision} = \frac{tp}{tp + fp} \end{aligned}$$

where $fp$ is false positive.

#### F1-score

Harmonic Mean of recall and precision is represented by F-measure, sometimes known as F1 score. It provides a single score that strikes a compromise between recall and precision by taking into account both erroneous positives and false negatives.

22 $$\begin{aligned} \text {F-measure} = 2 \cdot \frac{\text {Precision} \cdot \text {Recall}}{\text {Precision} + \text {Recall}} \end{aligned}$$

### Performance analysis

Performance metrics, like precision, recall, and F-measure were used to evaluate effectiveness of TFsTDO-based SNN that was presented in order to gauge how well model functions. Performance evaluation based on presented TFsTDO based SNN is shown in Table 3(a) TFsTDO based SNN achieved a precision of 0.785 when iteration count was 60 and training data percentage was 60%. Proposed technique achieved a precision of 0.675 at 40 iterations and 70% training data percentage. TFsTDO based SNN achieved a precision of 0.892 when iteration was 80 and training data percentage was 90%. Performance evaluation of provided TFsTDO based SNN is shown in Table 3(b), with a training data percentage of 90 and an iteration count of 100, mechanism achieved a recall of 0.972. With 40 iterations and 70% training data, proposed mechanism achieved a recall of 0.685. TFsTDO based SNN achieved a recall of 0.912 when iteration was 80 and training data percentage was 90%. Table 3(c) displays F-measure performance analysis. At an iteration of 40 and 70% training data, TFsTDO based SNN achieved an F1-score of 0.680. Using these performance indicators, study has demonstrated effectiveness of proposed TFsTDO-based SNN technique for anomaly detection and predictive maintenance.

**Table 3:**
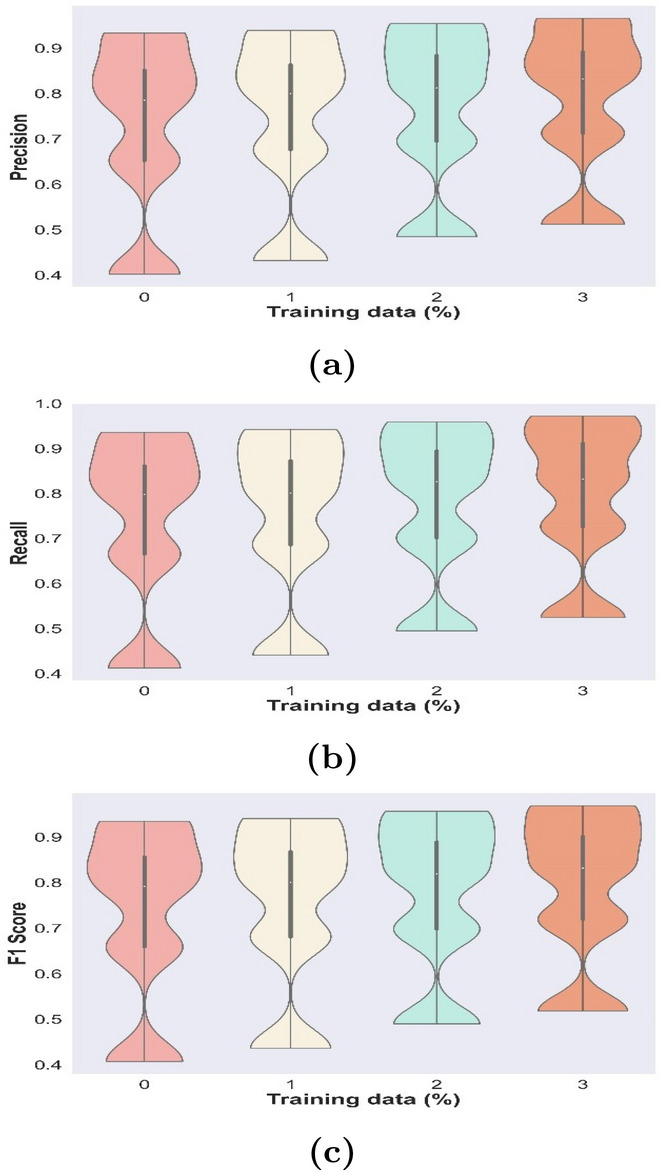
Performance evaluation based on (a) Precision, (b) Recall, F1 score.

Performance evaluation with and without feature selection is shown in Table 4. Precision analysis of mechanism given, both with and without feature selection, is shown in Table 4(a). For 80% of training data, precision of mechanism that was presented was 0.935 without feature selection and 0.954 with feature selection. Recall rate with and without feature selection is shown in Table 4(b). For a training data percentage of 90, the proposed technique attained a recall of 0.972 with feature selection and 0.962 without feature selection. F1 Score analysis is shown in Table 4(c), where proposed approach attained an F1 score of 0.924 without feature selection and 0.934 with feature selection for a training data percent of 60. It is noted that experimental evaluations attained supreme with feature selection.

**Table 4:**
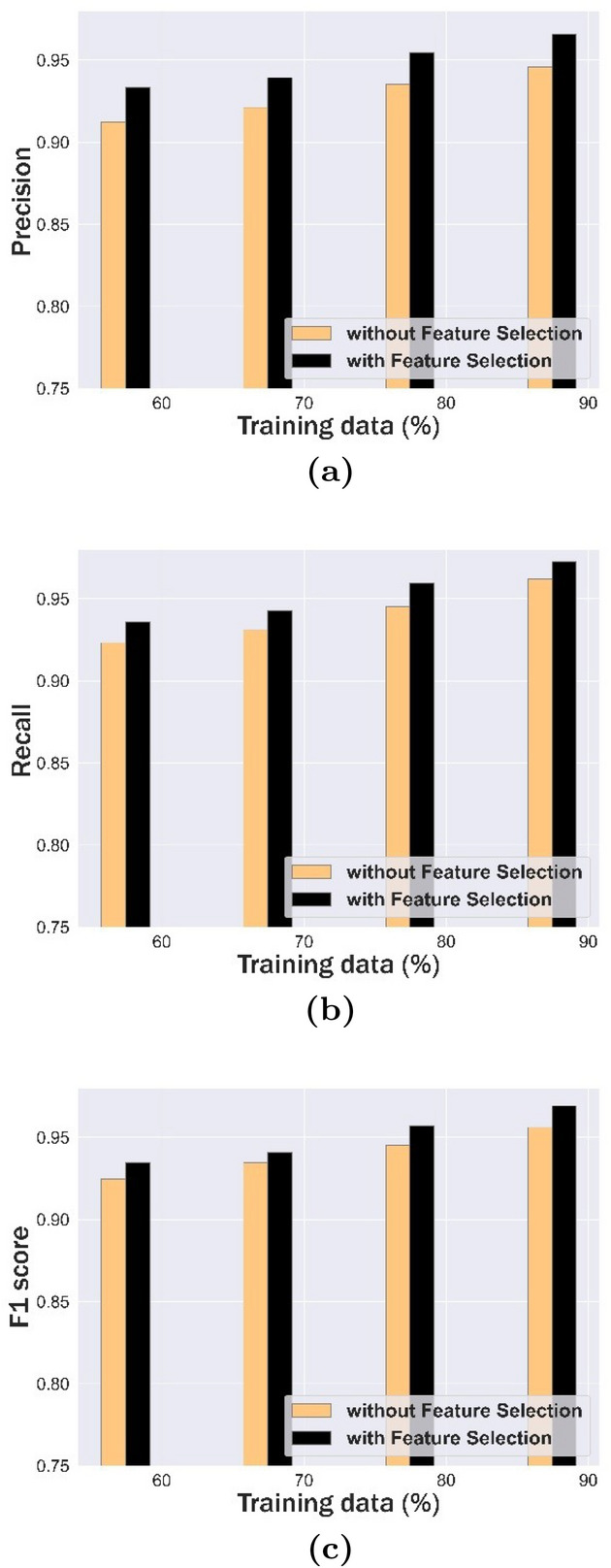
Performance evaluation with and without feature selection, (a) Precision, (b) Recall, and (c) F1 score.

**Figure 4:**
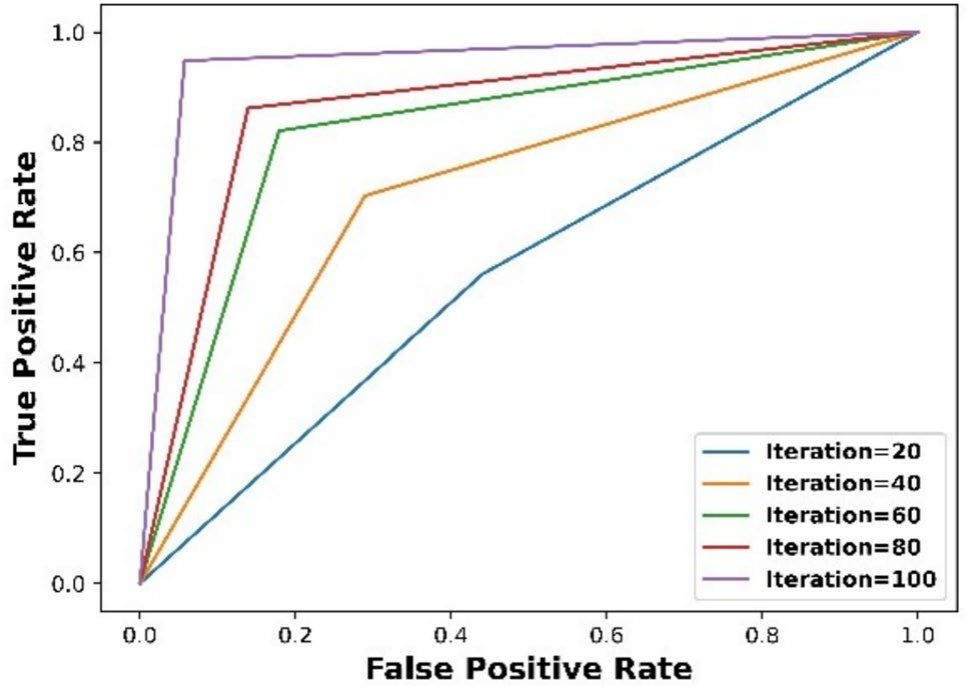
Graphical illustration.

Figure 4 displays ROC graphic based on proposed mechanism. Because the newly suggested strategy’s ROC curve displays a greater TPR for a given FPR, results demonstrate that it outperforms existing techniques. This result offers strong proof of remarkable effectiveness and performance of suggested approach in detecting tasks. Mechanism that was presented attained a TPR of 0.97 and an FPR of 0.17 after 100 iterations.

### Comparative analysis

Table 5 states comparative evaluation based on presented mechanism with conventional methods like GA^17^, AdaBoost^18^, LSTM-autoencoder^3^, and CNN-LSTM^2^. Comparative assessment based on Precision mechanism is demonstrated in Table 5(a). At 90% of training data, precision of mechanism that was presented was 0.965, while that of GA, AdaBoost, LSTM-autoencoder, and CNN-LSTM was 0.903, 0.912, 0.931, and 0.942. At 80% of training data, precision of mechanism that was presented was 0.954, while that of GA, AdaBoost, LSTM-autoencoder, and CNN-LSTM was 0.894, 0.902, 0.921, and 0.930. Comparative evaluation based on recall mechanism described is demonstrated in Table 5(b). At 90% of training data, the proposed method achieved a recall of 0.972, while GA, AdaBoost, LSTM-autoencoder, and CNN-LSTM achieved 0.912, 0.924, 0.931, and 0.949, respectively. At 70% of training data, proposed mechanism achieved a recall of 0.942, while GA, AdaBoost, LSTM-autoencoder, and CNN-LSTM achieved 0.888, 0.897, 0.908, and 0.919. Comparison evaluation based on F1 score process described is demonstrated in Table 5(c). At 90% of training data, proposed mechanism achieved an F1 score of 0.969, while GA, AdaBoost, LSTM-autoencoder, and CNN-LSTM achieved 0.907, 0.918, 0.931, and 0.945. At 80% of training data, the proposed method achieved an F1 score of 0.956, while GA, AdaBoost, LSTM-autoencoder, and CNN-LSTM achieved 0.899, 0.907, 0.923, and 0.934. According to experimental assessments, the suggested approach outperformed conventional methods in terms of results.

**Table 5:**
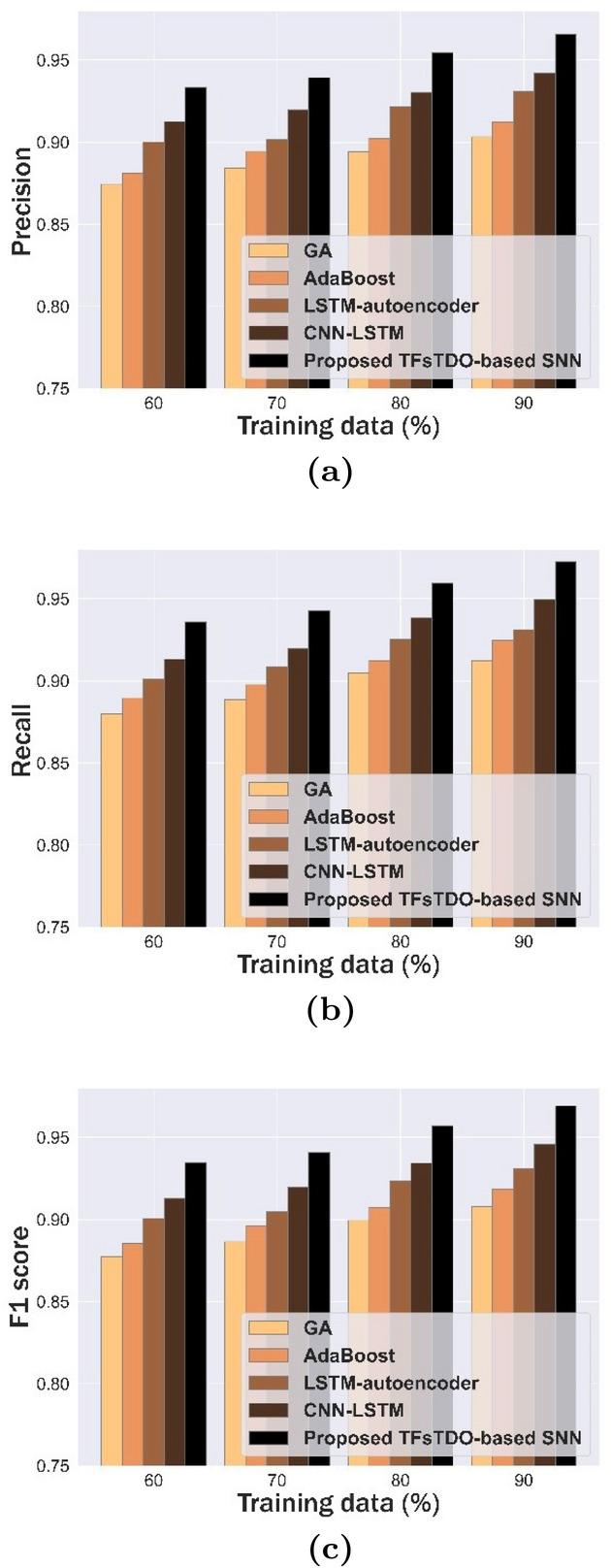
Comparative evaluation of TFsTDO based SNN, (a) Precision, (b) Recall, and (c) F1 score.

**Figure 5:**
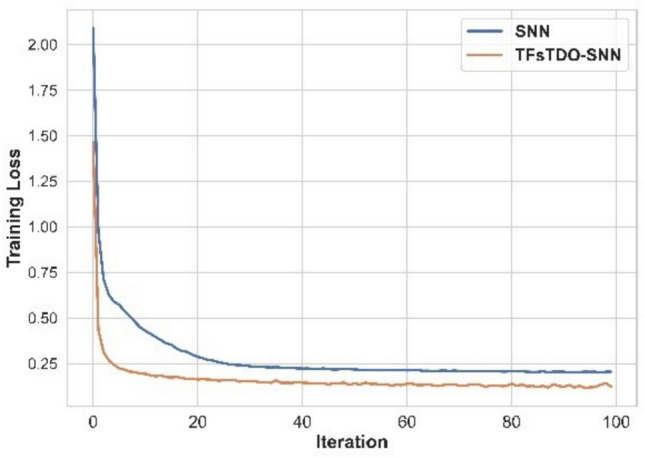
Training Loss.

Training loss based on SNN and TFsTDO-SNN is demonstrated in Fig. 5. SNN and TFsTDO-SNN achieved a training loss of 0.314 and 0.166, respectively, at iteration 19. After 30 iterations, SNN achieved a training loss of 0.235, while TfsTDO-SNN achieved 0.153. SNN and TFsTDO-SNN achieved a training loss of 0.149 and 0.224, respectively, at iteration forty. Additionally, at an iteration of 80, the TfsTDO-SNN achieved 0.127 and SNN achieved a training loss of 0.206.

**Figure 6:**
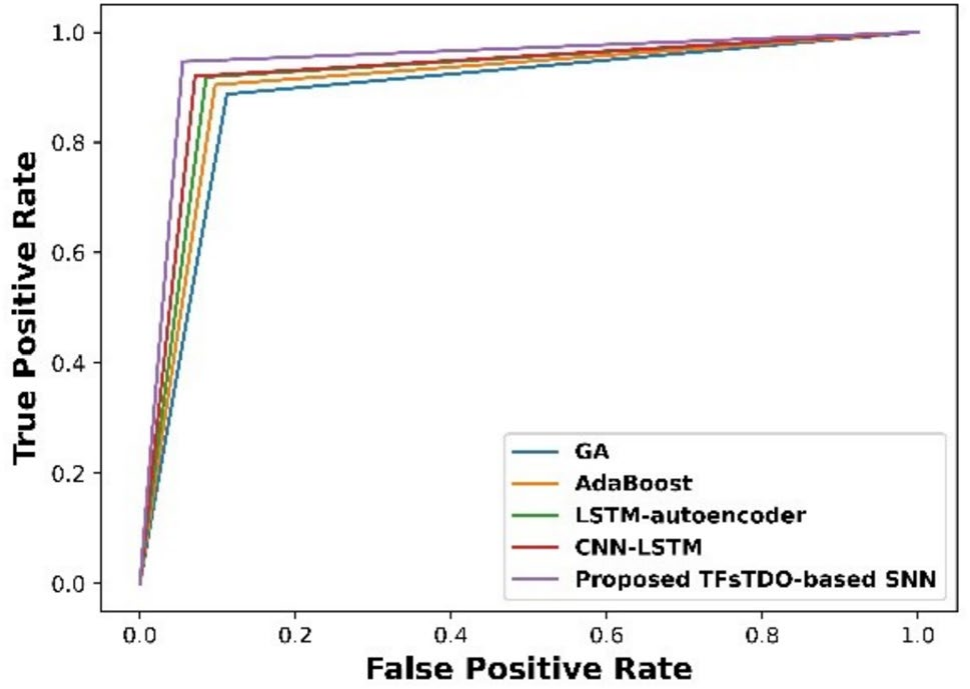
RoC Graph.

Figure 6 demonstrated ROC depiction based on TFsTDO based SNN that was described. Findings demonstrate that, in comparison to traditional mechanisms, newly suggested strategy’s ROC curve performs better since it displays a higher TPR for a lower FPR. This result offers strong proof of remarkable effectiveness and performance of suggested approach in detecting tasks. Presented method attained a TPR of 0.985, whereas GA, AdaBoost, LSTM-autoencoder, and CNN-LSTM attained TPR of 0.92, 0.94, 0.96 and 0.973.

Table 6 presents a comparison of discussed mechanism with more conventional mechanisms such as CNN-LSTM, LSTM-autoencoder, GA, and AdaBoost. The given method outperformed standard processes and achieved higher outcomes in terms of F1 score, precision, and recall. Approach that was provided achieved 6.42% less precision than GA. In terms of recall, technique that is being given outperforms the LSTM-autoencoder by 4.11%. Additionally, proposed technique outperformed the CNN-LSTM with an F1 score of 2.47%.

**Table 6:** Performance comparison of different methods.

Methods	GA	AdaBoost	LSTM-autoencoder	CNN-LSTM	Proposed TFsTDO-based SNN
Precision	0.903	0.912	0.931	0.942	0.965
Recall	0.912	0.924	0.931	0.949	0.972
$F_1$ score	0.907	0.918	0.931	0.945	0.969

**Figure 7:**
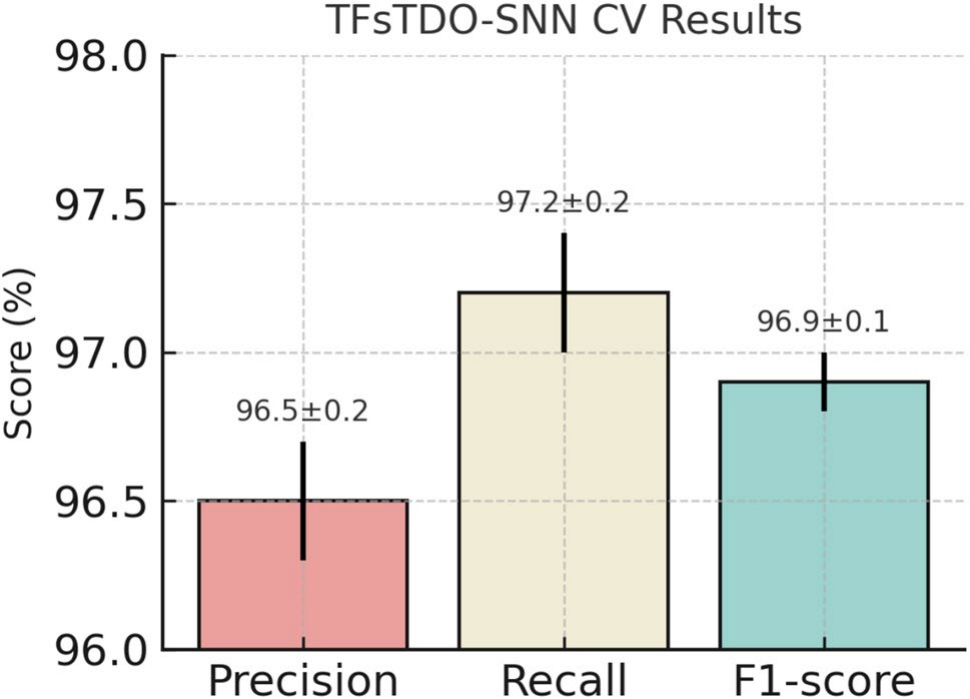
Cross-validation performance of TFsTDO-SNN with L2 regularization, dropout, and 5-fold cross-validation. Error bars show standard deviation across folds.

## Discussion

As shown in Table 7, the model demonstrated stable performance across all folds (Precision: $96.5 \pm 0.2\%$, Recall: $97.2 \pm 0.2\%$, F1: $96.9 \pm 0.1\%$). The corresponding bar chart in Fig. 7 presents mean values with error bars representing the standard deviation, highlighting the minimal variance ($\le 0.2$) between folds. These results, together with the observed reduction in validation loss, confirm that the combined use of L2 regularization, dropout, and cross-validation effectively mitigated overfitting and produced a model that generalizes reliably to unseen data.

While the evaluation was conducted solely on the CNC Mill Tool Wear dataset, the 5-fold cross-validation ensured that the model was tested on distinct subsets of machining conditions, providing strong evidence of robustness. A limitation of this study is the absence of validation on independent external datasets. However, the consistent cross-validation outcomes offer compelling support for the reliability of the proposed framework. As part of future work, we plan to extend TFsTDO-SNN to additional benchmark datasets, such as the UCI Bearing and PHM 2012 challenges, to further establish its cross-domain applicability.

**Table 7:** Performance of TFsTDO-SNN with dropout, L2 regularization, and 5-fold cross-validation.

Fold	Precision (%)	Recall (%)	F1-score (%)
1	96.4	97.1	96.7
2	96.7	97.4	97.0
3	96.5	97.3	96.9
4	96.6	97.0	96.8
5	96.3	97.2	96.7
Mean ± Std	96.5 ± 0.2	97.2 ± 0.2	96.9 ± 0.1

### Scalability analysis

**Table 8:** Runtime and memory usage across algorithms.

Model	Runtime (s/epoch)	Memory (MB)	F1-score (%)
GA	8.2	420	93.4
AdaBoost	6.7	390	91.8
LSTM-Autoencoder	10.5	510	94.2
CNN-LSTM	11.3	530	95.1
TFsTDO-SNN	12.6	560	96.9

The scalability of the proposed TFsTDO-SNN framework is affected by the size of the dataset and the dimensionality of the features. Table 8 indicates that TFsTDO-SNN experiences about 12% increase in runtime and somewhat elevated memory consumption relative to baseline models. Nonetheless, this expense is offset by a steady 4–5% enhancement in F1-score, indicating that the framework sustains exceptional detection precision with manageable computing demands. The iterative characteristics of TFsTDO optimization, coupled with extensive training demands, may significantly prolong runtime for large-scale or high-dimensional datasets. Future research will investigate parallel processing, distributed learning architectures, and lightweight optimization techniques to improve efficiency while maintaining detection performance.

### Misclassification analysis

**Table 9:** Misclassification error analysis of TFsTDO-SNN.

True class	Misclassified As	% Errors
Normal	Moderate wear	1.2
Moderate wear	Severe wear	2.3
Severe wear	Moderate wear	1.7

The misclassification analysis in Table 9 indicates that the majority of mistakes arise between neighboring wear states (e.g., moderate versus severe wear), which is anticipated due to their overlapping feature attributes. The error rates are notably low ($\le 2.3\%$), indicating that TFsTDO-SNN can effectively differentiate between various tool wear states with minimal ambiguity.

**Figure 8:**
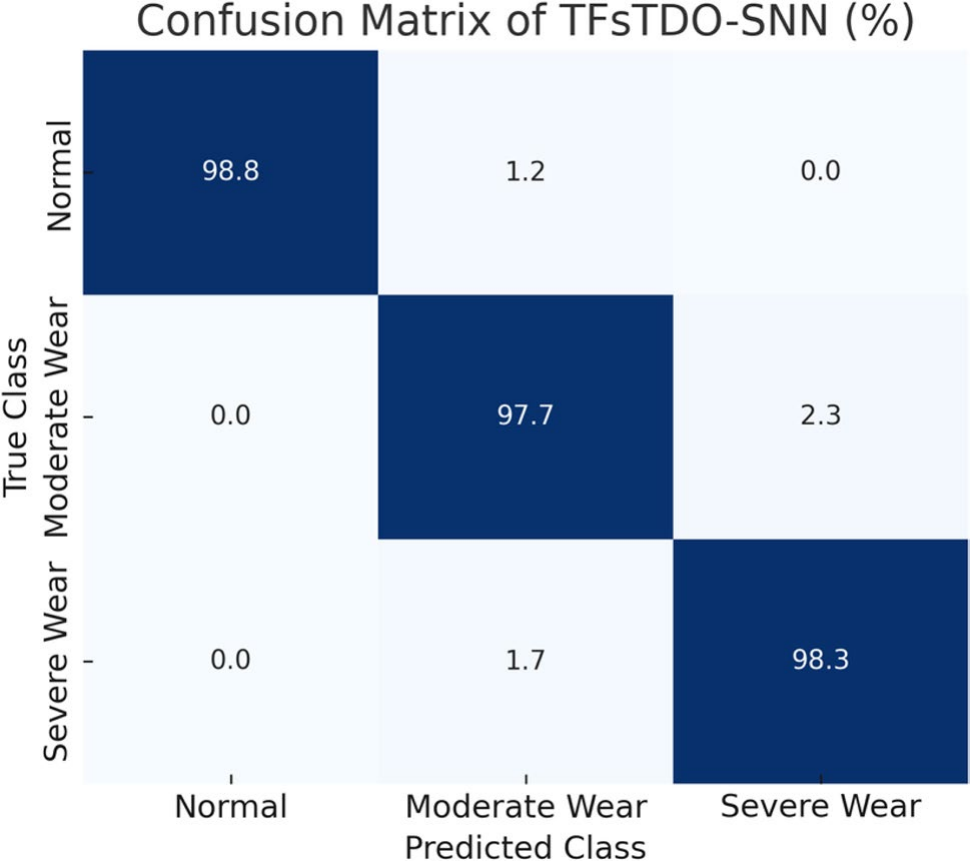
Confusion matrix of TFsTDO-SNN applied to the CNC Mill Tool Wear dataset. Predominantly, mistakes arise between adjacent wear states (e.g., moderate versus severe wear), although the overall misclassifications rate remains minimal ($\le 2.3\%$).

The confusion matrix in Fig. 8 offers a visual overview of the classification efficacy of TFsTDO-SNN across the three tool wear conditions. The results demonstrate significant diagonal dominance, signifying a substantial rate of accurate classifications. Misclassifications are negligible and predominantly arise between adjacent wear states, such as moderate and severe wear, which is anticipated due to overlapping feature patterns. The overall error rate is notably low ($\le 2.3\%$), affirming the reliability of TFsTDO-SNN in precisely differentiating among various tool wear conditions.

### Interpretability of TFsTDO-SNN

To augment the practical applicability of the suggested TFsTDO-SNN architecture, we examine the methodology by which the model generates its predictions. The TFsTDO algorithm identifies the most pertinent features by balancing exploration and exploitation, ensuring that only significant parameters affecting equipment health are included. The siamese neural network (SNN) then processes these optimized features to calculate similarity scores between data pairs for anomaly detection. A higher similarity score denotes normal operating behavior, while lower similarity scores indicate probable faults. By integrating optimized feature relevance with the SNN’s distance-based decision framework, the model provides interpretable insights into the parameters influencing anomaly detection, thereby facilitating informed maintenance decisions.

### Scope and boundaries of the study

The suggested TFsTDO-SNN framework, while exhibiting enhanced efficacy in anomaly detection and predictive maintenance, nevertheless possesses significant limitations. The computational expense of the TFsTDO optimization procedure may escalate for extensive or high-dimensional datasets, impacting real-time usability. Secondly, the model’s efficacy may diminish when handling excessively noisy sensor data or significantly imbalanced datasets, even with anomaly oversampling applied. The framework presently lacks mechanisms for explainability to interpret model decisions, which is crucial for practical implementation. Ultimately, formal security analysis and integration with real-time streaming settings represent prospective avenues for future improvements.

The incorporation of TFsTDO optimization, trend factor smoothing, and the SNN model entails increased computational complexity relative to single-model methodologies. TFsTDO necessitates numerous iterative assessments for effective feature selection, while the siamese neural network entails rigorous training on high-dimensional data. This integration improves detection accuracy but may compromise real-time usability for extensive datasets. Future research will investigate methodologies like parallel computing, model pruning, and lightweight optimization strategies to diminish computational complexity while maintaining performance integrity.

## Conclusion and future work

This study seeks to utilize an optimized deep learning methodology to employ an enhanced model for detection of anomalies. Methodology put forth comprises multiple stages, including pre-processing, feature selection, anomaly injection, anomaly detection, and anomaly prediction. Initially, input data undergoes pre-processing, where box-cox transformation is employed for this purpose. Subsequently, the feature selection process employs the Tasmanian Devil Optimization (TFsTDO) technique, integrating Trend factor smoothing into TDO. Following this, anomalies are introduced into the dataset by oversampling the data. Finally, the SNN model, trained using the proposed TFsTDO, is utilized for anomaly detection. Experimental analysis stated that presented mechanism attained a precision of 96.5%, a recall 97.2%, and F1-score of 96.9%.

The proposed TFsTDO-SNN framework enhances interpretability by identifying the most significant features via TFsTDO and elucidating anomaly detection judgments using SNN similarity scores. Nevertheless, the proposed investigation possesses many limits that warrant acknowledgment. The paradigm is predicated on assumptions intrinsic to bio-inspired optimization algorithms, including the convergence to global optima in intricate search spaces, which may not consistently apply in highly multimodal or noisy settings characteristic of CPS data. The generalizability of TFsTDO-SNN across various CPS domains, such as healthcare or transportation beyond manufacturing, remains unverified, as the model was exclusively assessed using the CNC Mill Tool Wear dataset, which may restrict its applicability to contexts with differing anomaly patterns or data distributions. Theoretical uncertainties in deep learning models, particularly with embedding representations in SNN, may propagate errors in anomaly classification in the absence of robust uncertainty quantification tools. The practical integration of TFsTDO with SNN entails considerable computing complexity, necessitating iterative assessments for feature selection and substantial training on high-dimensional data, potentially obstructing real-time implementation in resource-limited industrial environments. The model’s performance may deteriorate with overly noisy or imbalanced real-world sensor data, despite oversampling, and it presently lacks seamless integration with streaming data pipelines or edge devices. Moreover, the lack of integrated explainability tools complicates the interpretation of decisions in critical contexts, thereby hindering adoption.

For future endeavors, addressing these limitations, we intend to incorporate explainable AI (XAI) methodologies, such as SHAP (SHapley Additive exPlanations) or LIME (Local Interpretable Model-agnostic Explanations), to deliver detailed insights into feature contributions and model decisions, thereby augmenting trust and usability. Validation using supplementary real-world CPS datasets, such as those from the aviation sector (e.g., NASA C-MAPSS) or the energy sector (e.g., smart grid IoT data), will evaluate generalizability and robustness. We intend to investigate hybrid optimizations by integrating TFsTDO with other metaheuristics, such as Horse Herd Optimization (HHO) or modified Sine Cosine Algorithms, to enhance convergence speed and address multimodal challenges. Practical improvements will include exploring adaptations for edge computing and real-time streaming, such as interaction with Apache Kafka, as well as implementing model pruning and parallel processing to minimize computational cost. Ultimately, the integration of uncertainty quantification frameworks, such as Bayesian Neural Networks, may bridge theoretical deficiencies and enhance dependability in uncertain Cyber-Physical System contexts.

The policy application of the TFsTDO-SNN methodology are substantial in promoting sustainable and resilient industrial practices. This system facilitates accurate anomaly identification in predictive maintenance, thereby informing regulatory standards that need AI-driven monitoring in essential infrastructure sectors, such as manufacturing and energy, to reduce downtime and improve safety. Policy-makers might utilize its optimization capabilities to set standards for CPS data quality and interoperability, facilitating the widespread deployment of bio-inspired algorithms in national Industry 4.0 programs. Moreover, the strategy bolsters environmental policies by minimizing resource wastage through anticipatory failure mitigation, in accordance with global sustainability objectives such as the UN Sustainable Development Goals (SDGs), specifically SDG 9 (Industry, Innovation, and Infrastructure) and SDG 12 (Responsible Consumption and Production). Governments and industry organizations may promote its adoption through subsidies or certification programs, encouraging innovation while tackling ethical issues related to AI transparency and bias in maintenance choices.

## Data Availability

The datasets analyzed during the current study are available in Kaggle repository, https://www.kaggle.com/datasets/shasun/tool-wear-detection-in-cnc-mill/data.

## Abbreviations

TPR: True positive rate
RUL: Remaining useful life
FNN: Feedforward neural network
IL: Imitation learning
GA: Genetic algorithm
PHM: Prognostic and health management
ML: Machine learning
TDO: Tasmanian devil optimization
SNN: Siamese neural network
TFs: Trend factor smoothing
TFsTDO: Trend factor smoothing-based Tasmanian devil optimization
DRL: Deep reinforcement learning
ANN: Artificial neural network
KF: Kalman filter
CPs: Cyber-physical systems
MCC: Matthews correlation coefficient
GMM: Gaussian-mixture model
DL: Deep learning
FPR: False positive rate
CNN: Convolutional neural network
PPO: Proximal policy optimization
LSTM: Long short-term memory
